# Nanomaterials and Chemical Modifications for Enhanced Key Wood Properties: A Review

**DOI:** 10.3390/nano9040607

**Published:** 2019-04-12

**Authors:** Antonios N. Papadopoulos, Dimitrios N. Bikiaris, Athanasios C. Mitropoulos, George Z. Kyzas

**Affiliations:** 1 Laboratory of Wood Chemistry and Technology, Eastern Macedonia and Thrace Institute of Technology, GR-661 00 Drama, Greece; 2 Laboratory of Polymer Chemistry and Technology, Department of Chemistry, Aristotle University of Thessaloniki, GR-541 24 Thessaloniki, Greece; dbic@chem.auth.gr; 3 Hephaestus Advanced Laboratory, Eastern Macedonia and Thrace Institute of Technology, GR-654 04 Kavala, Greece; amitrop@teiemt.gr

**Keywords:** wood, nanomaterials, nanotechnology, lignocellulosic materials, chemical modification, acetylation

## Abstract

This work briefly reviews the research milestones in the area of wood chemical modification, focusing on acetylated and furfurylated wood which have been scaled up, and exploits the solutions that nanotechnology can offer to wood protection as an alternative green innovative approach in improving key wood properties, namely the dimensional stability when subjected to a fluctuating moisture content and a susceptibility to biodegradability by microorganisms. Recently, nanomaterials were found to be able applicable in wood science. The target is to improve some special physicochemical characteristics of wood in order to resist extreme conditions (climate, bacteria, etc.), giving an enhanced potentiality. It is well-established that the wood cell wall shows a porosity of molecular scale dimensions; this is caused by the partial filling of spaces between the microfibrils of the cellulose mainly by polyoses and lignin. The small-sized nanoparticles can deeply and effectively penetrate into the wood, altering its surface chemistry, improving its properties, and therefore, resulting in a hyper-performance product.

## 1. Introduction

Wood, a versatile material, has been used for centuries for many reasons due to its fibrous nature. It varies in color and density and is considered a primary raw material in buildings due to its high strength in combination with its low weight and some durability. It is, therefore, a raw material that can be used in indoor applications and, if treated efficiently, in outdoor application as well. However, wood has two main disadvantages which restrict its wider use, namely a susceptibility to biodegradability by microorganisms and a dimensional instability when subjected to a varied moisture content. Most wood species deteriorate rapidly under biological factors; the most important biological decay is caused by fungi. On the other hand, when wood is subjected to a fluctuating moisture, dimensional and conformational instability occur. These drawbacks are mainly due to the cell wall main polymers and, in particular, due to their high abundance of hydroxyl groups (OH) [1,2,3]. This is solved, so far, either by using imported tropical woods or by using conventional biocides which are usually based in the use of toxic chemicals. In addition, the disposal of the treated wood has caused many restrictions which have to be taken into consideration upon the utilization of conventional chemical treatments. However, both these options are nowadays under political and consumer pressure and alternatives have become an imperative need [1]. This need led to attention to non-biocide treatments, such as the modification of wood with nontoxic chemicals. By applying chemical modifications species of low durability, mainly acetylated and furfurylated wood, can be easily transformed to entirely new products with improved properties; this approach, therefore, can be considered as the ultimate solution to the problem [3,4].

Another option to overcome this problem, perhaps a more attractive one, is to investigate the potentials that nanotechnology may offer. It is well-established that the wood cell wall shows a porosity of molecular scale dimensions; this is caused by the partial filling of spaces between the microfibrils of the cellulose mainly by polyoses and lignin [5,6]. The small-sized nanoparticles can deeply and effectively penetrate into the wood, altering its surface chemistry, improving its properties, and therefore, resulting in a hyper-performance product.

Therefore, this work briefly reviews the research milestones in the area of wood chemical modification, focusing on acetylated and furfurylated wood which have been scaled up, and exploits the solutions that nanotechnology can offer to wood protection as an alternative green innovative approach.

## 2. Chemical Modification

The main target of applying chemical modifications onto wood is to alter the molecular structure of the main cell wall polymers, by converting the hydrophilic OH-groups into hydrophobic groups. This way, the cell wall of wood is in a permanently swollen situation and attracts no or very little moisture [7,8]. At the same time, the chemically modified wood is not recognized by the degrading fungi, since the lower moisture content does not promote decay. Various technologies in the field of wood modification have been developed, mainly in laboratory and on a semi-industrial scale since there was no economic or environmental urgency to scale up these technologies. Recently, due to environmental and legislative issues on the use of biocides, new avenues for the chemical modification have been created. Wood modification technologies may be categorized, related to the mode of action of the chemical, as follows [1,3]:

- Filling the lumen with a chemical, usually resin: This decreases the sorption of water vapor but the sorption behavior over a longer period of time is not reduced.
- Bulking the cell wall and the cell lumen with a chemical: This reduces the swelling of wood and affects positively the long-term sorption behavior.
- Modifying treatments: The chemistry of the main cell wall components is changed, and covalent bonds are formed by converting the hydrophilic OH-groups into hydrophobic groups. This results in new wood products with enhanced properties.

A chemical modification refers to the linking of a chemical to the main cell wall polymers of wood, especially the hydroxyl groups via a covalent bond [7]. Chemical modification has been extensively reviewed elsewhere [1,3,8,9,10]. Section 2.1 and Section 2.2 focus on the most prominent processes, namely, acetylation and furfurylation. Both processes have been scaled up to the industrial level.

### 2.1. Acetylation

**Figure 1:**
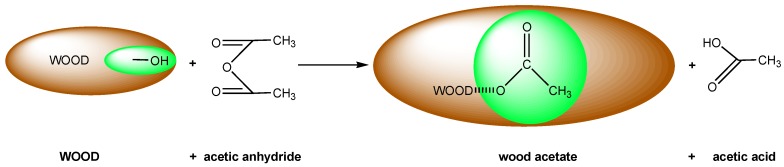
The reaction of wood with acetic anhydride.

A great amount of research in this area involves the reaction of anhydrides with wood. Anhydrides react with the hydroxyl groups of the wood cell wall polymers via a nucleophillic reaction pathway [11,12,13]. Researchers have mostly studied the reaction of hydroxyls groups with acetic anhydride, a process that is called acetylation. The reaction is depicted in Figure 1, where the wood hydroxyl groups are replaced by the acetyl groups of the acetic anhydride and the wood remains in a swollen condition due to the bulking action of the acetyl groups within the cell walls. Acetylation is a single site reaction; this means that one acetyl group is added per reacted hydroxyl group with no further polymerization. The produced products are wood acetate and acetic acid.

The first attempt of producing acetylated wood has been recorded in 1865, when Schutzenberger acetylated cellulose. Fuchs [14] and Horn [15], in the late 1920’s, were the first researchers who acetylated wood flour; whole wood was firstly acetylated by Stamm and Tarkow [16]. Since then, a lot of laboratories worldwide have tried to acetylate wood with a variety of ways [17]. The first attempt, however, to commercialize the process was not successful in the USA (1961), Russia (1977) and Japan (1984). On the semi-industrial level, the first successful scaled-up acetylation was performed at Stichting Hout Research (the Netherlands), by Prof. Militz and coworkers [18]. Nowadays, acetylated wood is scaled-up in the Netherlands, under the commercial name Accoya^®^, which utilizes radiate pine and alder wood with a 20% acetyl weight gain. Accoya^®^ wood undergoes a nontoxic acetylation process which modifies wood permanently.

#### 2.1.1. Water Vapor Sorption of Acetylated Wood

**Figure 2:**
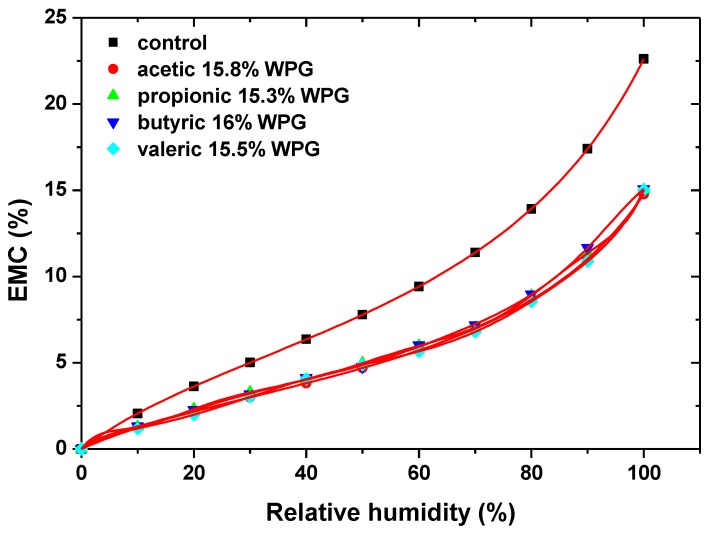
The adsorption isotherms for pine wood: control (■); acetic 15.8% WPG-weight percent gain (●); propionic 15.3% WPG (▲); butyric 16% WPG (▼); and valeric 15.5% WPG (◆) [21].

Many studies have examined the sorption of the water vapour of acetylated wood, and the overall stabilisation was well-documented [19,20]. A study by Papadopoulos and Hill investigated the effect that the molecular size of a substituent group may have on the sorption of water vapour, as wood was modified with a series of anhydride molecules of different sizes, which at the same time, produced equivalent levels of cell wall bulking at different levels of hydroxyl substitution [13,21]. The equilibrium moisture content (EMC) data were analyzed by applying the Hailwood–Horrobin sorption model, which enabled the separation of the total sorption into monomolecular and polymolecular sorption. They found that the reduction in all types of sorption was mainly a bulking effect and was not determined by the number of hydroxyl groups that had been substituted (Figure 2).

#### 2.1.2. Durability of Acetylated Wood

**Figure 3:**
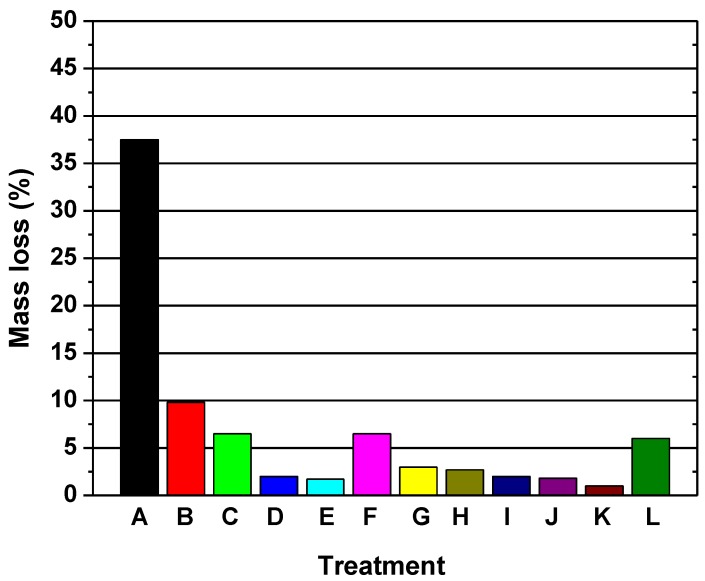
The mass loss (%) caused by the soft-rot decay of wood modified with linear chain anhydrides. A: untreated controls; B: acetic anhydride 7.5%; C: acetic anhydride 15%; D: acetic anhydride 15.5%; E: acetic anhydride 22.3%; F: propionic anhydride 10.2%; G: propionic anhydride 14.8%; H: propionic anhydride 20.5%; I: propionic anhydride 25.1%; J: butilic anhydride 23.3%; K: valeric anhydride 26.1; L: hexanoic anhydride 25.1%. Reprinted with permission from [27], Copyright Elsevier, 2010.

The resistance against fungi: The durability of acetylated wood against fungi and other microorganisms has been extensively studied in the literature; a level of modification of approximately 20% is required to achieve full protection [22]. The protection threshold levels for acetylated pine was determined by Beckers et al. [23]. They found that a weight gain of 18% was required against the brown-rot fungi, and a value over 20% was required against the white-rot fungi. A value of 20% is reported to prevent an attack by fungi in the ground contact stake tests [24]. Suttie et al. [25] reported that the bulking effect is the only factor that determines the protection from decay. Hill and Papadopoulos [26] reaffirmed this conclusion when they presented a comprehensive evaluation of the effectiveness of linear chain anhydrides against brown-rot fungi. This conclusion is further reaffirmed when pine samples were exposed to soft-rot decay [27], as depicted in Figure 3.

Resistance against marine wood borers and termites: Few studies have been published so far that focused on the protection against invertebrates [28] including marine wood borers. Papadopoulos et al. [29] used a laboratory test which was developed by Borges et al. [30] and assessed if the modification of pine wood with a series of anhydrides offers resistance to the crustacean wood borer *Limnoria.* The results revealed firstly that modification resulted in a significant reduction in the number of faecal pellets produced and, secondly, that the anhydride type had a minimal effect on feeding. 

A recent study has shown that pine wood modified with a series of anhydrides afforded significant protection in feeding above a 16% level of modification, against the subterranean termites [31].

### 2.2. Furfurylation

Stamm [32] has initiated research work relating to a chemical modification with furfuryl alcohol, known as furfurylation. Furfuryl alcohol is a liquid produced from agricultural wastes, such as sugar cane and corn cobs. Furfurylation is executed by impregnating wood with a mixture of furfuryl alcohol and catalysts and then heating it to cause polymerisation. The purpose of furfurylation is to improve the resistance to biological degradation and dimensional stability by applying a nontoxic, proprietary, furfuryl alcohol polymer. Furfurylation involves the impregnation of wood specimens with furfuryl alcohol and catalysts and a subsequent heating which, in turn, causes polymerisation [33]. The final product looks more like those of a common polymer-filled cell wall rather than a typical reacted cell wall [34]. The Norwegian company Kebony AS, applies the technology and produces two distinct products: (i) Kebony Clear, which is a hard furfurylated wood with a typical weight gain of 35%, and (ii) Kebony Character, a light furfurylated wood with a typical weight gain of 20%.

The industrial process of wood furfurylation is comprised of the following steps [34]:

- Storage and mixing of chemicals: The treating solutions are mixed in a separate mixing tank where different chemicals (furfuryl alcohol, initiators/catalysts, buffering agents, surfactants, and water) are added. The mixed solution is pumped to one of the buffer tanks.
- Impregnation: the wooden material, i.e., treatable softwoods or hardwoods, is vacuum pressure impregnated with the treating solution by a full-cell process with a vacuum step, a pressure step, and a short post-vacuum step.
- Reaction and curing: An in situ polymerisation of the chemicals and grafting reactions with the polymeric components of the wood occur during this step. The curing chamber is heated with a direct injection of steam, and the temperature achieved depends on the product use. The chamber is operated as a closed system during the curing period, except for a ventilation period at the end. The ventilation gas is cooled, and the condensate is separated from the gas; thereafter, the condensate goes back to the condensate tank for reuse.
- Drying: The final drying of the modified wood material in a kiln dryer is essential to minimise emissions and to obtain a desirable final moisture content.
- Cleaning: the emissions during the process are managed by cleaning the ventilated gases.

An excellent review on the properties of furfurylated wood was recently performed by Mantanis [34]. According to his findings,

- The biological durability of wood is up graded to “Class 1”. A decay test have showed that furfurylated wood of moderate loading has a comparable resistance with that of pine wood impregnated with copper chromium arsenate;
- The mechanical properties of wood, except for impact resistance, are enhanced when wood is treated with a furfuryl-alcohol polymer. Furfurylated wood is characterized by a greater hardness, elasticity, and rupture moduli as compared to untreated wood;
- Kebony wood, depending upon the loading, exhibits a strong dimensional stability and resistance to weathering; moreover, its water swelling and shrinking values are over 50% lower than untreated wood;
- Furfurylated wood is extremely resistant to marine borers at high levels (>50%) of weight percentage gain; and
- Studies regarding the ecotoxicology of furfurylated wood and leachates from furfurylated wood showed no significant ecotoxicity, while its combustion did not release any volatile organic compounds or polyaromatic hydrocarbons above the normal levels of wood combustion.

At this point, it has to be mentioned that natural products such as extractives, essential oils, and resins attract the researchers to develop environmentally friendly wood protectors. Lignin has attracted less attention as a wood preservative, whereas lignin derivatives seem to have a potential for the development of environmentally friendly biocides [35]. The importance of lignin utilization is frequently pointed out within the frame of biorefinery concepts. Kraft lignin as a by-product of the pulping industry is especially attractive for the development of value-added products because of its availability and low molecular mass [36,37].

At the same time, biotechnology including the application of enzymes and fungal cultures found its way also to the pulping and paper industry. Particularly, laccases are enzymes widely used to functionalize lignocellulosic materials. The application of enzymes in wood protection is seldom investigated. In a recent study, Kraft lignin (KL) showed apparent antifungal properties in Petri dish cultures [38]. In another study, the potential of the grafted KL for enhancing wood durability was assessed [39]. Two different wood treatments with KL was proposed and assessed: First, KL was enzymatically attached to mini-blocks of Scots pine and beech wood alone, and second, the same approach was applied in the presence of copper. The protective effectiveness against wood-destroying basidiomycetes was evaluated by accelerated aging tests. It was found that KL grafting in combination with copper entrapment improved the decay resistance and that the copper leaching was reduced.

## 3. From Nanotechnology to Nanomaterials

At this point, it is mandatory to explain why nanotechnology and, especially, nanomaterial are so necessary in the above proposed technology (wood protection). The American Nobel Prize-winning physicist Richard Feynman, in a speech in 1959, expressed the view that there is nothing in the laws of physics to prohibit the layout of people at will, arguing, “I am not afraid to think that the ultimate goal is to be able, in the distant future, to treat people exactly the way we want”.

Feynman argued that the only obstacle to the arrangement and handling of the matter at both the molecular and atomic level was the lack of experimental organs on a nanoscopic scale. Moreover, the laws of physics do not make any restrictions. Moving to ever smaller sizes, there is a need to redesign our experimental arrangements, as the correlation of forces changes. On this scale, the van der Waals forces and the surface tension rather than gravity play a more important role.

In a sense, nanotechnology is not entirely a new concept. A large amount of chemical substances or, in some cases, chemical processes have special properties at the nanoscale level. For example, for decades, chemists have synthesized polymers, large molecules structured by microscopic subunits. Nanotechnology has also been used for over twenty years to construct microscopic features in computer chips. The life around us also presents examples of nanoscale structures; the most classic example is that of milk which is a colloidal nanoscale or even some highly synthesized proteins which can play an important role in biological activities (the release of energy, cell repair, etc.). It is fact that nanoparticles exist in a natural way around us and have been formed for many years (thousand). An example in real life to confirm the above is the combustion products and food cooking.

Eigler and Schweizer proposed a unique technique to synthesize, adjust, produce, or even manipulate nanoparticles/nanomaterials; in general, all those techniques can be classified as “top-down” or “bottom-up”: (i) the “bottom-up approach”, which is based on the creation of devices using the fundamental components of matter, namely atoms [3], and the (ii) “top-down approach”, in which the construction of devices is based on cutting and etching [4]. These two methods have evolved separately and have now reached the point where the best possible feature size for each technique is about the same, thus leading to new hybrid modes of manufacture.

**Figure 4:**
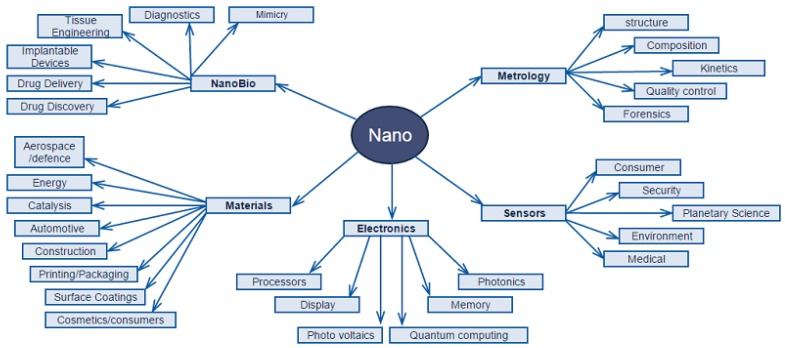
Applications in nanoscale.

Nanotechnology is considered to be a genuinely interdisciplinary field because it encourages cooperation between researchers who previously belonged to different fields, sharing knowledge, tools, and techniques. Therefore, physics, chemistry, medicine, biology, engineering, and too many relevant subsciences belong on the nanoscale level. Indeed, it could be argued that progressive developments in each of these areas for explorations on an ever-smaller scale have now come to be known under the term “nanotechnology”. It is widely known that nanotechnology is the combination of the technology on the nanoscale level. This major advantage makes nanotechnology a huge field with numerous subsections (the so-called “sub-applications”) (Figure 4).

Nanomaterials are already used for commercial purposes in industry. The nowadays available commercial products belong to a broad technologic area, including stain-resistant and wrinkle-free textiles, cosmetics, sunscreens, electronics, paints, and varnishes. Nanocoatings and nanocomposites are finding uses in diverse consumer products, such as windows, sports equipment, bicycles, and automobiles [40]. There are novel UV-blocking coatings on glass bottles which protect beverages from damage by sunlight and longer-lasting tennis balls using butyl-rubber/nano-clay composites [41]. Nanoscale titanium dioxide, for instance, is finding applications in cosmetics, sunblock creams, and self-cleaning windows, and nanoscale silica is being used as a filler in a range of products, including cosmetics, dental fillings [41], and other applications [42,43,44].

### Historical Points

The big bang was very crucial for nanomaterial science given that many theories refer to the existence of nanostructures after the fall of early meteorites. After that start, nature physically created some other nanostructure-like formations such as seashells, skeletons, etc. However, the scientific/technological story and the official start of nanomaterials can be pinned much later. Some important time-points for nanomaterial development are as follows:

(i) 1857: The synthesis of colloidal gold particles by Michael Faraday.
(ii) 1930s: Some novel catalysts in nanostructure form have been synthesized.
(iii) 1940s: Precipitated and fumed silica nanoparticles were developed and manufactured for applications as substitutes for ultrafine carbon black for rubber reinforcements. The target was the USA and German markets.
(iv) 1960s–1970s: Magnetic recording tapes were developed by metallic-like nanopowders.
(v) 1976: The inert-gas evaporation for nanocrystal synthesis was mentioned for first time by Granqvist and Buhrman.
(vi) Nowadays, the development of engineering nanophase/nanostructures or, generally, the development of nanotechnology is very rapid, and too many novel structural materials (either organic or inorganic) are investigated. Apart from that, we can also find possible ways to slightly or deeply modify/treat some useful parameters of nanomaterials (mechanical, optical, electronic functions, etc.).

## 4. Nanotechnology and Wood

Nanotechnology, an attractive science, seems to have a remarkable potential in producing new generation materials with enhanced properties [45]. The term “nano” is usually referred to materials with dimensions less than 100 nm. Nanomaterials have a high surface–volume ratio that enables them to show greater activities in surface-related phenomena compared to bulky systems with an identical mass [46,47,48,49,50].

The change in material properties is primarily due to the large interfacial area, which is developed per unit of volume, since the level of added particles is reduced to nanometers. Nanomaterials enhance the properties of the original material, show a great compatibility with the traditional materials, and cause a limited alteration of their original features [51,52,53,54,55].

Nanotechnology is being developed for a great variety of applications, from medical uses to material behavior improvement, like wood and wood products. Studies on the application of nanotechnology in wood science are mainly focused on the dimensional stability and resistance to attack by microorganisms. The main advantage of applying nanotechnology in wood science is the unique ability of the nanoparticles to penetrate deeply into wood substrates [56,57,58,59,60]. It is well-established that the cell wall of wood shows a porosity of molecular scale dimensions [5,6,57]. The small-sized nanoparticles can easily, effectively, and deeply penetrate into the wood, to alter its surface chemistry, and to improve its properties, therefore resulting in a hyper-performance product. In addition, a complete penetration and a uniform distribution can be achieved if the size of the nanoparticles is smaller than the diameter of the pits in the wood cell wall [61].

Currently, nanomaterials are applied to wood and is performed with three different and distinctive ways, namely (i) through the direct impregnation of nanosized materials into wood (nanosized metals), (ii) with the controlled release of nanomaterials embedded in a nanocarrier (polymeric nanocarriers), and (iii) by coating treatments [62].

### 4.1. Nanosized Metals

Nanosized metals can be basically synthesized using mainly two chemical approaches, namely (i) solution-based synthesis (sol-gel, sonochemical, and solvothermal) and (ii) vapour-based synthesis (combustion and chemical vapour deposition) [61,62,63,64,65,66]. It is well-documented that the method of synthesis determines the physicochemical characteristics of the nanosized metals [64]. Their main unique characteristics are a high surface to volume ratio, a uniform size distribution of the particles, and a great stability [45,56,67].

Their use in wood has the objective to enhance its physico-mechanical properties and its durability against microorganisms, since it is generally acceptable that nanosized metals may interact with the bacterial elements, leading gradually to cell death [53,67] or even to a disruption of the enzyme function [52,67]. A common practice is to disperse the nanosized metals in an organic polymer resin in order to form a nanocomposite. A key factor in this process is the proper dispersion of nanoparticles in order to obtain maximum improvements in wood properties. It is important to examine how well the metals interact with a wood substrate, since big deviations may cause phase separation and final material failure. An accumulation of the nanosized materials due to the high surface energy that they possess, on the other hand, can result in the formation of micro-composites in the wood substrate that may behave as a weak zones for potential stresses, especially at higher loadings [68]. Nanosized materials like metal nanoparticles (gold, copper, and silver) and metal oxides (zinc and aluminum) are nowadays widely applied to confer wood protection.

As far as copper is concerned, while traditional alkaline copper preservatives are solubilized in aqueous solution, nano-copper compounds are initially dispersed in water, and subsequently, the suspension is used for the treatment of wood. A fixation of nano-copper compounds primarily occurs through the deposition on cell wall layers and in pits [69,70]. It was reported that, in some cases, fungi may be not be able to recognize copper nanoparticles. Once nanoparticles enter the fungal cell wall, they form a reactive oxygen species with the fungus cell. In addition, nanoparicles may also undergo dissolution and, thereby, interfere with homeostatic processes within the fungal cell [71,72]. Akhtari and Nicholas exposed wood to a termite attack and found that nano-copper formulation could reduce the weight loss from 46.8 to 0.2% [73]. Treating wood with nano-copper oxide in the presence of polystyrene improved the dimensional stability [74].

The interest in silver nanoparticles for different types of applications has grown worldwide. This type of nanoparticle has also gained popularity in the improvement of wood properties and has been the subject of numerous studies. The possibility to improve the sorption behaviour of wood by applying a nano-silver-based compound was studied by Mantanis and Papadopoulos [75]. The compound reduced the total sorption of water vapor to a great extent. Similar results were also reported elsewhere [76,77]. Silver nanoparticles were applied to improve the durability in a series of tropical series [77]. In all cases, the treated woods were classified as highly resistant for white decay fungi, as opposed to untreated wood, of which the weight losses were more than 20%.

Zinc-based nano-compounds have also been applied to wood to improve its durability [78]. It was found that the mass loss due to fungi and termite attacks was significantly inhibited by the zinc-based preparations. In an another study, lime wood treated with a zinc-based nano-compound had about a 56% higher resistance against to *Trametes versicolor*—a white-rot fungus—and a 40% higher resistance against the *Coniophora puteana*—a brown-rot fungus [79,80].

**Figure 5:**
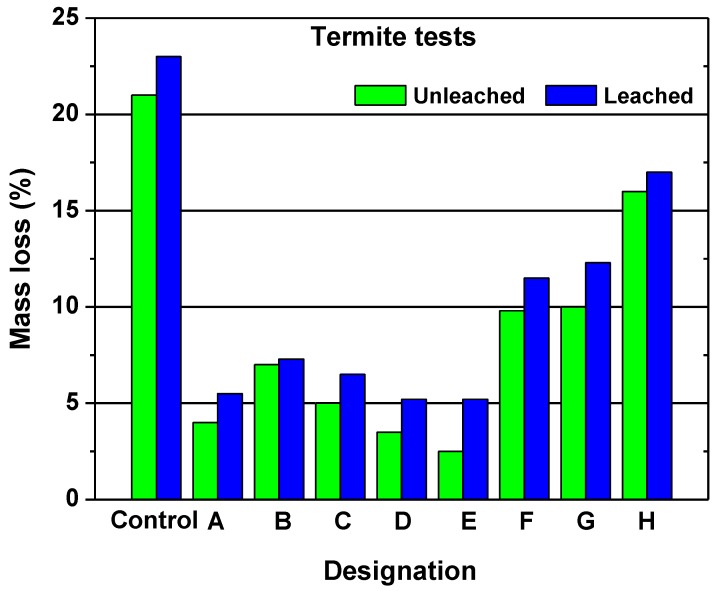
The mass loss (%) following termite resistance for leached and unleached pine wood: (**A**) nanozinc oxide; (**B**) nanozinc oxide plus binder A; (**C**) nanozinc oxide plus binder B; (**D**) nanozinc borate; (**E**) nanozinc borate plus binder A; (**F**) nano-copper oxide; (**G**) nano-copper oxide plus binder A; and (**H**) nano-copper oxide plus binder B. Reprinted with permission from [78], Copyright Elsevier, 2014.

**Figure 6:**
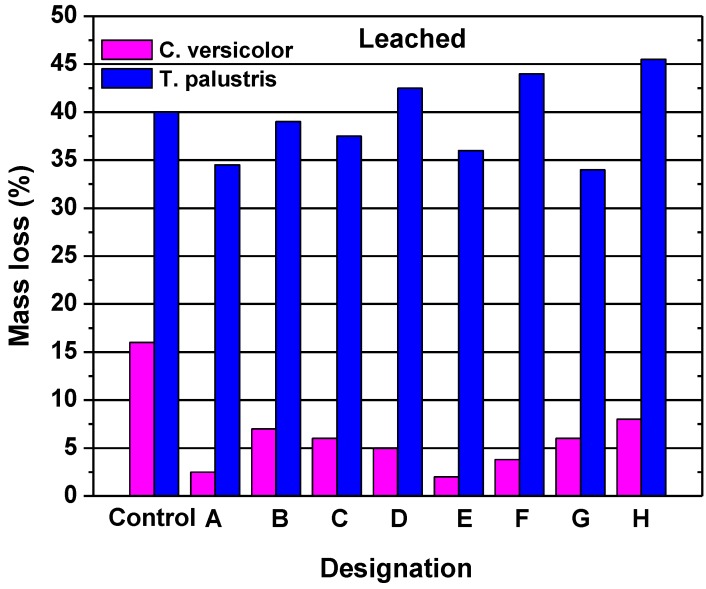
The mass loss (%) following a decay test for leached pine wood: (**A**) nanozinc oxide; (**B**) nanozinc oxide plus binder A; (**C**) nanozinc oxide plus binder B; (**D**) nanozinc borate; (**E**) nanozinc borate plus binder A; (**F**) nano-copper oxide; (**G**) nano-copper oxide plus binder A; and (**H**) nano-copper oxide plus binder B. Reprinted with permission from [78], Copyright Elsevier, 2014.

In other studies, nano-compounds based on zinc and copper have been applied to examine the resistance against mold and termite [78,81]. The results revealed that the compound based on zinc is more effective in terms of termite mortality, the inhibition of termite feeding, and the decay by the white-rot fungus (Figure 5 and Figure 6). Nano-compounds based on zinc seem to be most appropriate for wood protection [82,83]. Bak and Nemeth [84] studied the efficacy of five different nanoparticles, namely (zinc-oxide, zinc-borate, copper borate, silver, and copper) against fungi. They found that the most effective nanoparticle treatments were those containing borate. However, only the zinc-oxide, silver, and copper nanoparticles showed a resistance to leaching, and therefore, they concluded that only the zinc-oxide provided effective protection after leaching.

Titanium dioxide (TiO_2_ or titania), another nanomaterial, is a photocatalyst which is activated by ultraviolet light [85]. Furthermore, titania nanoparticles are used as a surface treatment which allows a thin water film to be formed on the treated substrates [86,87]; this reduces the water availability and prevents biological growth.

### 4.2. Polymeric Nanocarriers

**Figure 7:**
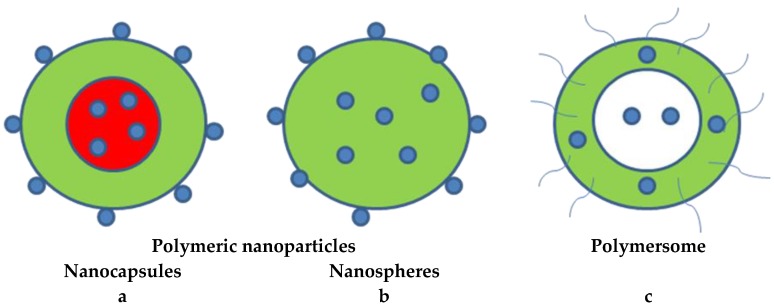
Types of polymeric nanocarriers: (**a**,**b**) The polymeric nanoparticles where active ingredients are conjugated to or are encapsulated in polymers. (**c**) A polymersome composed of hydrophilic-hydrophobic block copolymers, arranged in a lipophilic bilayer vesicular system and with a hydrophilic inner core.

Polymeric nanocarriers are also present in the wood preservation industry in improving the impregnation with traditional preservatives; however, they received far less attention in researches. An encapsulation of the active ingredient into polymeric nanocarriers can be conducted through several techniques such as nanoprecipitation [88]. Figure 7 depicts various types of polymeric nanocarriers that may be used for an active ingredient delivery [62,88,89]. 

The incorporation of organic biocides into polymeric nanoparticles and the subsequent introduction of the nanoparticles into wood may be advantageous. Conventional biocides, even those with a low solubility, can be easily dispersed in a solid polymeric nanoparticle that can be, in turn, suspended in water and, then, can be applied into wood with conventional water-borne treatments. With this way, biocides with a particularly low solubility that, so far, had a limited portion in the market may be now found a place in the wood preservation industry. In this case, the polymer wood substrate will act as a storage reservoir, which will control the release rate of the biocide, and at the same time, it will protect the unreleased biocide from exposure to the environment. This may cause a protection of the material for a long term [90].

Fungicides, such as chlorothalonil were incorporated into polymeric nanoparticles with a great success [90]. Polyvinylpyridine and polyvinylpyridine-co-styrene can be employed as the polymer matrices. Wood treated with biocide-containing nanoparticles showed great resistance against the white-rot fungi *Gloeophyllum trabeum*.

Nanotubules are other potential nanocarriers that can be a promising material for use as a carrier for biocides due to their hollow structure and high contact surface area. Carbon nanotubules (CNTs) are one of the best known nanotubules, which offer several advantages that include a high carrying capacity, a high biocompatibility, and a high surface area to volume ratio.

### 4.3. Coating Treatments

Nanotechnology may be applied in wood coatings in order to offer further improvements or even to result in new performance properties. Fillers, such as silicate-based minerals, are commonly used in coatings in order to increase the mechanical performance, mainly the hardness and stiffness, of a polymer matrix or to minimize cost. Their application in wood, when they are micron size, has two main disadvantages: It reduces the flexibility of the material and decreases the transparency of the coating system [68,91].

Polymeric coatings filled with nanoparticles such as spheres or tubes can be used to improve wood properties. Nanosized materials like metal nanoparticles are widely used to achieve these improvements and can be incorporated to the coating formulations by two different ways, through solution blending (physical approach) or in situ (chemical approach) [62,68]. The first approach involves an addition to the polymer in an appropriate solvent and then the application of forces to achieve dispersion. The nano-based coating is then applied on a wood surface by spraying, brushing, or dipping [75,85]. An in situ addition involves the addition of the nano-compound directly to monomers, dispersing them and polymerizing the mixture. The applied nanomaterials are in situ synthesized on a wood surface by chemical reactions like sol-gel deposition and hydrothermal methods. The chemical approach seems to be beneficial since smaller molecules can easily diffuse between nanoparticles, resulting therefore in an easy separation.

A popular coating material is the development of hydrophobic surfaces on wood; in this case, the nanomaterials act as water repellents and dimensional stabilizers. For example, beech wood treated with zinc-oxide nanoparticles provided a substantial water resistance and dimensional stability [92].

Nano-TiO_2_ [93] and nano-ZnO [94,95] are also capable of removing the microorganisms from surfaces, but they achieve this through their photocatalytic behavior by the production of reactive-free radicals (previously described) which can then attack and kill the microorganisms. However, at the same time, this can degrade the polymeric binder, so this mechanism cannot be so advisable for wood coatings. Nanozinc borate is also capable of reducing microbial activity [96], and although the mechanism may not be completely understood, it was previously related to the morphology of its crystals [97]. It has also been documented that borate increases the formation of reactive oxygen species which is analogous to the behavior of nano-TiO_2_ and -ZnO [98].

Another promising nanoparticle is nanosilica. An advantage of this nanoparticle is that it can be easily modified to improve the compatibility with polymers. It is found to reduce water absorption and to improve UV absorption characteristics [93]. Wu [99] has measured the influence of nanosilica on the UV curing of epoxy acrylic coating. They studied the influence of nonmodified nanosilica and silica modified with an acryl silane that contained tertiary amine function. At a 2% dosage, both types of silica not only absorbed UV light and increased the induction time of the initiator but also improved the diffusion of growing polymer chains, which led to an increased final conversion. Grenwood [100] investigated the influence of a high loading of colloidal nanosilica on several crylic and polyurethane resins. Changes in the properties differed, but as a general trend, there was a little increase of gloss. Hardness usually increased with less time being needed to reach final hardness. The abrasion resistance was either better or worse, and they correlated this with the level of dispersion. Vu [101] examined the influence of the base coat on the properties of the entire (base coat/topcoat) coating system. Colloidal nanosilica was added to the topcoat, which had an expected influence on the mechanical properties, such as improving the abrasion resistance and scratch resistance.

An improvement of the UV absorption characteristics can be also be achieved by the use of nano-clays [94]. These are silicates with a platelet structure and a high aspect ratio. This combination produces a road for molecules that have to track a longer route through the coating. As a result, the water absorption of the material was reduced, and the UV absorption characteristics were improved. Substantial work was done by Landry [102,103,104], who investigated the influence of different dispersing techniques commonly used in the coating industry (high speed disperser, ball-mill, bead-mill, and three-roll mill) on nano-clay composites in UV-curable epoxy acrylic lacquers. Malucelli [105] compared the behavior of nano-clay with boehmite, an aluminum oxide hydroxide mineral constituted of double layers of aluminum-centered oxygen octahedra which are held together by hydrogen bonds [106,107]. From the results of the rheological studies, the authors suggested there was little interaction between the fillers and the polymer, but the thermal properties and permeability were improved, especially with nano-clay, which was expected because of its layered platelet structure.

Nanocellulose is another nanofiller which looks like fiber or rod-like crystals. Its elongated structure offers a potential for stress transfer and can also provide a high reinforcement of binders. Semicrystalline nanofibrillated cellulose (NFC) is produced through mechanical shearing, while nanocellulose crystals (NCC) are produced by acid hydrolysis; in this way, the amorphous regions are removed [108,109].

Furthermore, nano-based coatings with strong bacterial properties, such as silver, zinc-oxide, copper, and titanium dioxide nanoparticles, also can provide decay resistance to wood and wood products and can, therefore, reduce the microbial activity. When these types of materials are considered for use in a coating treatment, the controlled and slow release of the active ingredient is important due to its prolonged positive effect and its minimal environmental impact [68].

## 5. Advantages of Nanomaterials in Wood Treatment

Nano-based treatments provide wood products with a better performance than conventional wood treatments due to its easy penetration and distribution, dispersion stability, and low viscosity properties. These treatments can improve the scratch and abrasion resistances, the blocking of UV radiation, the reaction to fire properties, and the hygroscopic properties without affecting the appearance of the wood. Apart from modification of existing properties, nano-based treatments also introduce new behaviour such as self-cleaning and photocatalytic properties. A variety of nanomaterials including nanoxides (TiO_2_, ZnO, SiO_2_, and CeO_2_), metal nanoparticles (Ag and others) and nano-clays have been used in wood modification either in their pure form or in combination with different additives as discussed in previous section. In the following table (Table 1), the basic advantages of nanomaterial use are presented compared to the major disadvantage.

## 6. Conclusions

This article briefly reviewed the research milestones in the area of wood chemical modification, focusing on acetylated and furfurylated wood which have been scaled up, and exploited the solutions that the science of nanotechnology can offer to wood protection as an alternative, green innovative approach. It is well-established that the wood cell wall shows a porosity of molecular scale dimensions due to the partial filling of spaces between the cellulose microfibrils mainly by polyoses and lignin. The small-sized nanoparticles can deeply and effectively penetrate into the wood, alter its surface chemistry, and improve its properties, resulting therefore in a hyper-performance product. In addition, a complete penetration and a uniform distribution can be achieved if the size of the nanoparticles is smaller than the diameter of the pits in the wood cell wall. Currently, nanomaterials are applied to wood and is performed with three different and distinctive ways, namely through the direct impregnation of nanosized materials into wood (nanosized metals), with a controlled release of nanomaterials embedded in a nanocarrier (polymeric nanocarriers), and by coating treatments. The change in material properties is primarily due to the large interfacial area that is developed per unit of volume, since the level of added particles is reduced to nanometers. Nanomaterials enhance the properties of the original material, show a great compatibility with the traditional materials, and cause a limited alteration of their original features. Their use in wood has the objective to improve its physical properties, mainly its hygroscopicity and its resistance against mold, decay fungi, marine borers, and subterranean termites. Furthermore, an increased UV protection may also be achieved.

## Figures and Tables

**Table 1:** The advantages of nanomaterials in wood preservation versus conventional treatments.

Property	Advantage	Disadvantage
Hydrofobicity	Nanocomposite coatings create rough hydrophobic surface without affecting the softness and abrasion resistance of the wood. The impregnation of nanomaterials reduces the pore size and space available within the cell wall, which is used for the absorption of water molecules.	Although nanotechnology is widely integrated in wood treatment, recently there are increasing critiques and discussions concerning the potential health and environmental risks of nanomaterials. Past experience shows that new technologies may not be more environmentally superior than traditional ones either due to the possibility of shifting the negative impacts outside the impact category or even the late identification of their impacts. Even if the incomplete knowledge or a lack of information about nanomaterials delays the study, addressing the potential impact of nano-based treatments using tools like a life-cycle assessment will help to avoid mistakes like in the past when new technological innovations were released prior to an impact assessment.
UV-protection	A surface modification with nano-sized inorganic fillers has found many applications as they are nontoxic and stable under exposure to UV. Their large surface to volume ratio makes them effective towards improving poor the photoresistance property of the wood.	
Fire performance	Wood is composed of cellulose, hemicelluloses, and lignin, which can make it undergo thermal degradation when it is in contact with a source of ignition. Although the fire performance of wood is improved using different fire retardants, there have been a low efficiency, leaching, and a high environmental and health risk while utilizing many of these chemicals. The utilization of nanoparticles alone or in combination with other fire-retardant chemicals can reduce the ignitability of the wood and limit the leaching of fire-retardant chemicals.	
Antimicrobial	Nanoparticles increase the decay resistance of wood by reducing the moisture availability in the wood either by preventing the absorption of the moisture or by blocking the flow path of liquid water.	
Mechanical properties	The mechanical properties of wood depend on environmental agents, its isotropic properties, and sometimes on the type of treatment used to improve wood attributes. Impregnating the wood with nanoparticles enhances its hardness by filling the cavities of the wood.	
